# GPER and EGFR cross-talk highlight aldosterone- and MR antagonist-induced NO production in cultured endothelial cells

**DOI:** 10.3389/fphar.2026.1891002

**Published:** 2026-09-01

**Authors:** Renata Paredes, M. Verónica Donoso, Carlos F. Lagos, César Cárdenas, Claudio Acuña-Castillo, Daniel Valdes, Luis Constandil, Juan Pablo Huidobro-Toro

**Affiliations:** 1 Laboratorio de Farmacología, Departamento de Biología, Facultad de Química y Biología, Universidad de Santiago de Chile, Santiago, Chile; 2 Chemical Biology & Drug Discovery Lab, Escuela de Química y Farmacia, Facultad de Ciencias, Universidad San Sebastián, Santiago, Chile; 3 Centro Basal Ciencia & Vida, Fundación Ciencia & Vida, Santiago, Chile; 4 Centro Biología Integrativa, Facultad de Ciencias, Universidad Mayor, Santiago, Chile

**Keywords:** aldosterone, GPER/GPR30, mineralocorticoid receptor antagonists, endothelial nitric oxide, vascular reactivity, molecular docking

## Abstract

**Introduction:**

Aldosterone induces rapid, non-genomic vasodilation of the rat mesenteric vasculature, and mineralocorticoid receptor (MR) antagonists are widely used in cardiovascular disease, yet their pharmacological profiles beyond classical MR blockade remain unclear. We examined whether the G protein-coupled estrogen receptor (GPER/GPR30), functionally coupled to the epidermal growth factor receptor (EGFR), accounts for the rapid endothelial nitric oxide (NO) component of aldosterone action, and whether MR antagonists display intrinsic GPER-linked activity.

**Methods:**

Aldosterone-induced vasodilation was assessed in isolated perfused rat mesenteric arterial beds precontracted with noradrenaline, and NO production was quantified in primary mesenteric endothelial cell cultures by DAF-FM fluorescence with validated vehicle controls. The mechanisms engaged by spironolactone, eplerenone and finerenone were interrogated with selective pharmacological tools and by endothelium removal. Ligand recognition was explored by molecular docking in a comparative model of the rat GPER built on the human GPER cryo-EM template.

**Results:**

Aldosterone elicited concentration-dependent endothelial NO production (EC_50_ = 2.26 ± 0.3 nM) and vasodilation (EC_50_ = 0.9 ± 0.2 nM) that were closely correlated (R^2^ = 0.969, p < 0.01). All three MR antagonists blocked the aldosterone responses, yet each also evoked intrinsic, concentration-dependent NO production in the absence of aldosterone, eplerenone and spironolactone showing greater intrinsic efficacy than finerenone. Pretreatment with 100 nM of the GPER antagonist G-36 or 100 nM AG-1478 significantly reduced both aldosterone-induced DAF-NO signaling and vasodilation, whereas endothelium removal or L-NAME converted vasodilation into vasoconstriction. Aldosterone signaling additionally required PI3K, PKA and IP_3_ receptor-gated Ca^2+^ mobilization. Docking identified plausible receptor-engaging poses, with steroidal ligands converging on a shared cavity and finerenone adopting a distinct binding mode.

**Discussion:**

MR antagonists elicit endothelial NO production through a G-36-sensitive, GPER-linked pathway functionally coupled to EGFR signaling, rather than acting as pure competitive antagonists in this context. Because recent cryo-EM work reveals a non-canonical extracellular GPER architecture, the docking results are interpreted as structurally plausible interaction scenarios rather than definitive orthosteric assignments; whether the behaviour reflects direct partial agonism at GPER or an indirect, GPER-dependent mechanism remains to be established by direct-binding studies. These findings expand current understanding of MR antagonist pharmacology.

## 1 Introduction

Aldosterone, the principal mineralocorticoid hormone, plays critical roles in cardiovascular homeostasis beyond its classical genomic effects on renal sodium retention (Gomez-Sanchez and Gomez-Sanchez, 2014; Funder, 2017). Over the past 2 decades, accumulating evidence has demonstrated that aldosterone also elicits rapid, non-genomic cardiovascular effects occurring within seconds to minutes, independent of gene transcription or protein synthesis (Funder, 2005; Michea et al., 2005; Grossmann and Gekle, 2009). These rapid effects include vasodilation (Liu et al., 2003; Heylen et al., 2009), modulation of vascular tone (Schmidt et al., 2003), and alterations in endothelial function (Romagni et al., 2003; Mutoh et al., 2008). Collectively, these findings establish nitric oxide (NO) as a central mediator of aldosterone’s rapid vascular effects.

The G protein-coupled estrogen receptor (GPER, previously known as GPR30) has emerged as a key mediator of rapid, non-genomic steroid signaling (Revankar et al., 2005; Prossnitz and Barton, 2011; Prossnitz and Arterburn, 2015). GPER is a seven-transmembrane receptor that activates diverse signaling pathways including PI3K/Akt, MAPK/ERK, and calcium mobilization (Maggiolini and Picard, 2010; Filardo and Thomas, 2012). While initially characterized as an estrogen receptor, accumulating evidence suggests GPER responds to multiple steroid hormones linked to rapid cardiovascular effects (Haas et al., 2009; Meyer et al., 2011).

**FIGURE 1:**
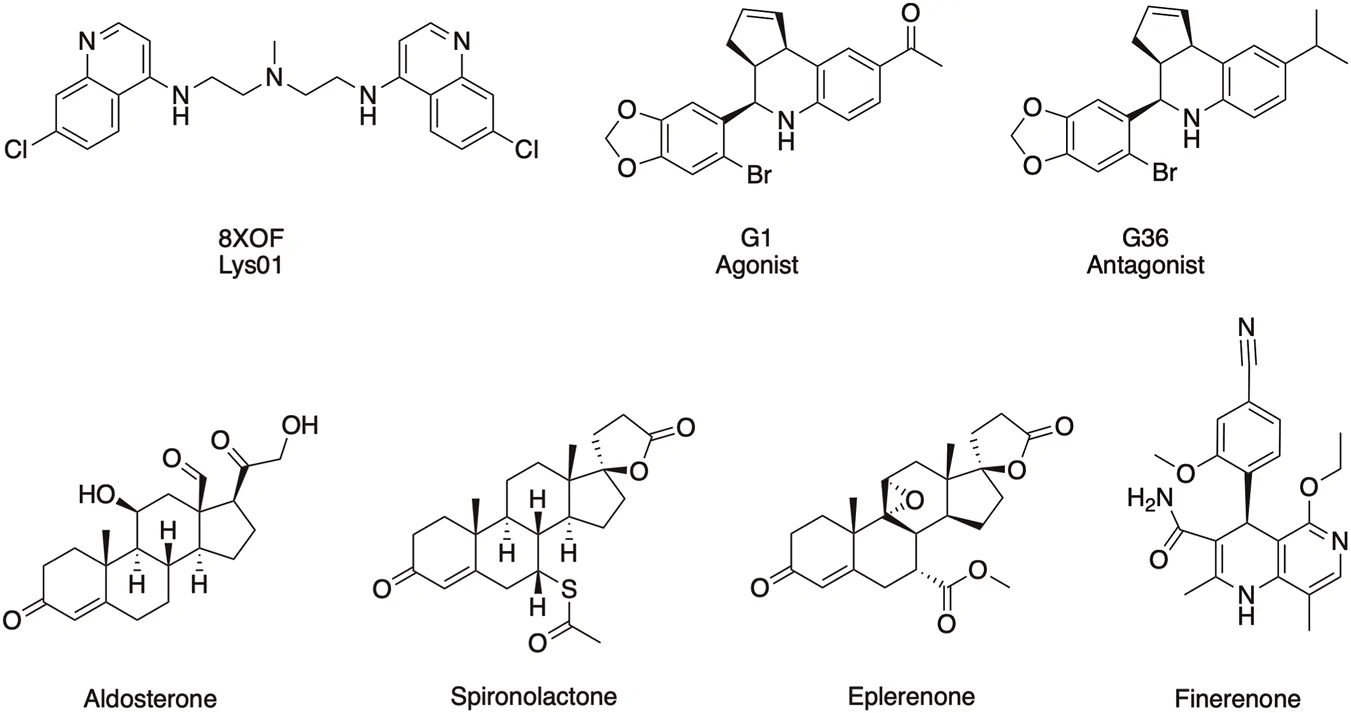
Molecular Structures of GPER and MR modulators. Chemical structures of known GPER and mineralocorticoid receptor ligands. G-1, G-36 and Lys01 are reported GPER ligands. Aldosterone, spironolactone, eplerenone, and finerenone showing structural similarities (steroidal compounds) and differences (non-steroidal finerenone). Steroidal MR antagonists (spironolactone, eplerenone) share greater structural similarity with aldosterone than non-steroidal finerenone, potentially explaining differences in partial agonism efficacy.

The development of selective GPER agonists and antagonists has been instrumental in delineating GPER’s physiological roles. The synthetic agonist G-1 (1-[4-(6-bromobenzo [1,3]dioxol-5-yl)-3a,4,5,9b-tetrahydro-3H-cyclopenta [c]quinolin-8-yl]-ethanone) was identified in 2006 as a GPER-selective compound with minimal activity at classical estrogen receptors ERα and ERβ (Bologa et al., 2006). G-1 has been extensively used as a research tool and has demonstrated therapeutic potential in preclinical models of cancer, metabolic disease, and cardiovascular disorders (Arterburn and Prossnitz, 2023; Prossnitz and Barton, 2023), chemical structure is shown in Figure 1. Building on the success of G-1, the enantiomerically pure compound LNS8801 has been developed for clinical translation, representing the first GPER-selective drug candidate (Natale et al., 2025). LNS8801 exhibits potent anticancer activity in preclinical models, including induction of mitotic arrest, upregulation of p53 and p21, promotion of cellular differentiation, and induction of apoptosis. In uveal melanoma xenografts, LNS8801 significantly inhibited tumor growth *in vivo*, demonstrating oral bioavailability and GPER-dependent efficacy (Ambrosini et al., 2023).

Recent cryo-electron microscopy (cryo-EM) studies have fundamentally impacted our understanding of GPER structure and ligand recognition. Kaneda et al. (2024) reported the first cryo-EM structure of human GPER at 3.2 Å resolution, revealing unique extracellular pockets and identifying bicarbonate ions as bound ligands. This groundbreaking work demonstrated that GPER possesses structural features distinct from classical steroid receptors, with specific residues critical for bicarbonate binding and receptor activation. Complementing this finding, structural and functional evidence challenging the long-held assumption that estrogen is the direct cognate GPER ligand, suggesting otherwise that GPER may function as an ion-sensing receptor with non-canonical ligand recognition mechanisms has been provided (Liu et al., 2024). The hGPER structure revealed a distinctive extracellular architecture in which the upper transmembrane region and extracellular loops define three extracellular pockets, designated A-C, rather than a classical deep steroid-binding cavity. Pocket A is the largest and most hydrophilic region and has been proposed as the principal bicarbonate-responsive site, whereas pockets B and C are smaller and more superficial (Kaneda et al., 2024). The identification of bicarbonate-binding pockets and the structural divergence in G-protein coupling geometry compared to other class A GPCRs, suggest that GPER activity may involve alternative endogenous ligands or indirect modulation by steroids through membrane-proximal mechanisms. This structural framework provides a foundation for interpreting GPER’s involvement in aldosterone’s rapid vascular effects and for understanding how synthetic GPER modulators interact with this receptor. Importantly, despite the non-canonical extracellular architecture revealed by cryo-EM, direct radioligand binding evidence has confirmed aldosterone as a *bona fide* GPER agonist. Using [^3^H]2-methoxyestradiol displacement assays in Sf9 insect cells engineered to express GPER, it was demonstrated that aldosterone binds GPER with a Ki of 22 nM. Functional studies confirmed correlation between binding affinity and biological activity, definitively establishing aldosterone as a direct GPER ligand (Ding et al., 2022). The physiological relevance of GPER in bicarbonate sensing and vascular protection has been further demonstrated *in vivo*, where bicarbonate signaling via GPR30 regulates ischaemia-reperfusion injury (Jo-Watanabe et al., 2024). Collectively, GPER has emerged as a promising therapeutic target for aldosterone-induced hypertension and cardiovascular disease (Hall and Filardo, 2023; Li et al., 2023).

Earlier homology modeling and molecular dynamics studies established the computational framework for GPER ligand interactions, including identification of key binding residues and characterization of agonist versus antagonist binding modes (Arnatt and Zhang, 2013; Méndez-Luna et al., 2015). Comparative modeling of inactive and active GPER states revealed dual binding modes for agonists and identified helix movements critical for receptor activation (Arnatt and Zhang, 2013). Furthermore, natural GPER ligands such as oleuropein and hydroxytyrosol have been shown to activate GPER/GPR30-dependent pathways (Chimento et al., 2014), expanding the repertoire of GPER-active compounds beyond synthetic pharmacological tools. Molecular docking and dynamics simulations provide insights into the binding modes of G-1 and LNS8801, with reported strong binding affinities for G-1 (−29.87 kcal/mol) and LNS8801 (−28.09 kcal/mol) to GPER, with Arg253 and Arg254 identified as key amino acid residues for binding these activators (Elamin et al., 2024). Computational studies revealed that GPER residues Arg, Glu, His, and Asn form hydrogen bonds with hydroxyl, ether, or amine groups of ligands, with G-1 forming hydrogen bonds with Glu121 (Lu et al., 2023). These structure-activity relationships constitute the bases for rational design of next-generation GPER modulators with improved selectivity and improved pharmacokinetic properties. Selective GPER antagonists, including G-15 and G-36, have also been developed to distinguish GPER-mediated effects from classical estrogen receptor signaling. G-36 exhibits more potent antagonism than G-15 in modulating calcium mobilization and PI3K signaling (Dennis et al., 2009; Dennis et al., 2011). Chemical structures are shown in Figure 1. These pharmacological tools have been essential for establishing GPER’s role in various physiological and pathophysiological processes, including the rapid vascular effects of aldosterone investigated in the present study.

A prominent feature of GPER signaling is its role to transactivate epidermal growth factor receptor (EGFR), thereby engaging downstream pathways such as PI3K/Akt and ERK. This receptor cross-talk has been documented in several cellular systems and may be especially relevant for endothelial responses that culminate in eNOS activity and NO production (Prossnitz et al., 2008). However, the contribution of GPER-EGFR signaling to rapid aldosterone responses and to the pharmacology of MR antagonists has not been fully clarified.

Mineralocorticoid receptor (MR) antagonists are cornerstone therapies for heart failure, hypertension, and chronic kidney disease (Pitt et al., 1999; Pitt et al., 2003). The steroidal MR antagonists spironolactone and eplerenone, and the non-steroidal antagonist finerenone, differ in their chemical structures (Figure 1), receptor selectivity profiles, and clinical efficacy (Kolkhof and Bärfacker, 2017; Agarwal et al., 2021). While these agents are classified as competitive MR antagonists, their potential for off-target effects and potential partial agonism at other receptors has not been fully investigated. Partial agonism, a phenomenon where an antagonist evidences intrinsic activity at a receptor, has important therapeutic implications. Partial agonists can produce submaximal responses even in the absence of the endogenous agonist, and their effects depend on receptor reserve, tissue context, and signaling pathway (Mailman, 2007; Kenakin, 2014). Recognition of partial agonist activity is critical for understanding the full pharmacological profiles of MR antagonists and their differential clinical effects.

Based on these premises, the present investigation was designed to: (1) characterize aldosterone-induced endothelial NO production and subsequent vasodilation in isolated rat mesenteric arterial beds; the correlation between these parameters was characterized (2) study the effects of three clinically used MR antagonists (spironolactone, eplerenone, finerenone) on aldosterone NO production and assess putative intrinsic activity; (3) determine the roles of GPER and EGFR in aldosterone-induced NO production using selective pharmacological antagonists; and (4) elucidate the intracellular signaling pathways mediating aldosterone’s rapid effects. (5) Assess through bioinformation tools the putative binding sites of the MR antagonists to GPER and calculate tentative binding affinities to the modeled rGPER. The present findings highlight that MR antagonists elicit endothelial NO production through a GPER-linked pathway, mediated by cross-talk between GPER-EGFR signaling pathways. Clinical pharmacological implications outlined from present results will support the putative development of next-generation MR modulators.

## 2 Materials and methods

### 2.1 Animals

Male Sprague-Dawley rats (250–300 g) were obtained from the animal facility of Universidad de Santiago de Chile. Animals were housed under standard conditions (12-h light/dark cycle, 22 °C ± 2 °C, food and water *ad libitum*) and handled according to protocols approved by the Institutional Animal Care and Use Committee. All procedures conformed to the Guide for the Care and Use of Laboratory Animals (NIH Publication No. 85–23, revised 1996).

### 2.2 Isolated perfused rat mesenteric arterial bed

Rats were anesthetized with a mixture of ketamine and xylazine (75 and 5 mg/kg, i.p.); upon deep anesthesia, the superior mesenteric artery was cannulated *in situ*. The mesenteric arterial bed was excised, mounted in a perfusion chamber, and perfused at constant flow (2 mL/min) with Tyrode buffer (mmol/L: 118 NaCl, 5.4 KCl, 2.5 CaCl2, 1.2 KH_2_PO_4_, 1.2 MgSO_4_, 23.8 NaHCO_3_ and 11.1 D-glucose), maintained at 37 °C and gassed with 95% O_2_/5% CO_2_. Perfusion pressure was continuously monitored using a pressure transducer connected to a Grass model 5 recording polygraph to assess continuously perfusion pressure. Perfusion pressure variations in noradrenaline precontracted mesenteries were monitored during a 3–4 h period of a protocol that followed strictly the perfusion characteristics as previously described (Calfío et al., 2021). After a 30-min equilibration period, preparations were preconstricted with 50 μM noradrenaline to achieve stable increased baseline perfusion pressure. Aldosterone or other test compounds were perfused into the perfusion line; changes in perfusion pressure were recorded continuously. Vasodilation was expressed as percentage decrease from the noradrenaline-induced baseline perfusion pressure.

### 2.3 Primary endothelial cell culture

Rat mesenteric endothelial cells were isolated by collagenase digestion as previously described (Donoso et al., 2018; Calfío et al., 2021; Catalán-Salas et al., 2024). Briefly, mesenteric arteries were dissected, cleaned of connective tissue, and incubated with collagenase type II (1 mg/mL) for 60 min at 37 °C. Endothelial cells were collected by gentle agitation, centrifuged, and cultured in M-199 media containing antibiotics, 20% fetal bovine serum plus 20 μg/mL endothelial cell growth factor supplements. Endothelial cell identity was confirmed by positive immunostaining for von Willebrand factor and CD31 as previously shown by Calfío et al. (2021). Endothelial cells were seeded in 25 mm diameter covers according to the Catalán-Salas et al. (2024) protocols and placed in the cell incubator maintained with controlled 5% CO2 pressure until approx. 3 days, a time necessary for the cells to reach circa 80% confluence. Thereafter, the cells were ready for the several predetermined protocols outlined for each experiment.

### 2.4 NO determinations

Single cell intracellular NO production was measured using the fluorescent indicator 4-amino-5-methylamino-2′,7′-difluorofluorescein diacetate (DAF-FM DA, Molecular Probes). Cells were washed with Tyrode buffer supplemented with 10 mM HEPES, pH 7.3 and loaded with 4-Amino-5-Methylamino-2′,7′-Difluoroescein Diacetate (DAF-FM diacetate, DAF) as (5 μM) for 30 min at 37 °C (Balcerczyk et al., 2005). After washing, cells were incubated with test compounds for 30 min, and fluorescence was measured using a Zeiss confocal microscope as detailed by Calfío et al. (2021) and Catalán-Salas et al. (2024); the microscope is equipped with a 488 Laser with a 40-fold amplification; frames were detected every 15 s for a total of 10 min. When the first minute was reached, the cells were stimulated by adding a 10 μL drop of Tyrode-HEPES buffer containing the ligand of interest. Background fluorescence from wells without cells was subtracted. To validate DAF-NO fluorescent specificity for NO, parallel experiments included the use of the NOS inhibitor L-NAME (100–300 μM) to support the enzymatic NO origin.

### 2.5 Drugs and chemicals

Aldosterone, spironolactone, eplerenone, noradrenaline, L-NAME (Nω-nitro-L-arginine methyl ester), wortmannin, H-89, and DAF-FM diacetate were purchased from Sigma-Aldrich. Finerenone was obtained from MedChemExpress. AG-1478 (EGFR inhibitor) was from Calbiochem. G-36 (GPER antagonist) was synthesized as previously described or obtained from Tocris Bioscience (Dennis et al., 2009). Desmethyl xestospongin C was synthetized in the lab associated to Dr. J.C. Cárdenas as reported by Catalán-Salas et al. (2024). Aldosterone, MR antagonists and other drug solutions were prepared in DMSO (final DMSO concentration ≤ 0.1% v/v) or ethanol and diluted in buffer immediately before use. Vehicle controls confirmed that solvents at these concentrations had no effect on measured parameters. Salts used to prepare Tyrode buffer were analytical degree purchased from Merck (Darmstadt, Germany).

### 2.6 Experimental protocols

#### 2.6.1 Concentration-response curves; NO production and mesentery vasodilatation

Non-cumulative concentration-response curves for aldosterone (0.1 nM - 1 μM) were constructed in both isolated mesenteric beds and cultured endothelial cells. In antagonist studies, preparations were pre-incubated with antagonist for 15 min before aldosterone addition. For MR antagonists’ intrinsic activity assessment, concentration-response curves for spironolactone, eplerenone, and finerenone (1–10 μM) were obtained in the absence of aldosterone. GPER antagonism was examined using 100 nM G-36 and the involvement of EGFR was assessed via preincubation with 100 nM AG-1478 for 30 min, agent which selectively inhibits EGFR phosphorylation and signaling.

#### 2.6.2 Signaling pathway studies

Inhibitors were selected to interrogate each proposed signaling node with the most widely validated, mechanism-specific pharmacological tool available: wortmannin (100 nM), an irreversible covalent inhibitor of the p110 catalytic subunit of PI3K, was used to test PI3K/Akt involvement; H-89 (10 µM), a competitive ATP-site inhibitor of the cAMP-dependent protein kinase (PKA) catalytic subunit, was used to test PKA involvement; and desmethylxestospongin B (dmXeB, 10 μM), a selective, membrane-permeant blocker of the inositol-1,4,5-trisphosphate (IP3) receptor, was used to test IP3-receptor–gated Ca^2+^ mobilization. Concentrations were chosen to match those previously validated for GPER-linked endothelial signaling under identical recording conditions (Catalán-Salas et al., 2024). The alignment between each pathway target, the chosen inhibitor and concentration, its mechanism of action, and the corresponding residual aldosterone-induced NO signal is summarized in Supplementary Table S2. To assess GPER and EGFR involvement, cells were pre-incubated with 100 nM G-36 or 100 nM AG-1478, respectively. It should be noted that at least 3–4 replicates were performed per protocol using cells harvested from separate animals.

#### 2.6.3 rGPER molecular modeling

As a starting point we first generated a comparative model of rat GPER (Uniprot id O08878). Blast search identified the cryo-EM structure of human GPER in complex with Lys05 (PDB id (XOF) as template (Liu et al., 2024). 100 molecular models of rGPER residues 45 to 349 including a disulfide bridge between cysteines 130–207 were generated using Modeller v10.2 (Webb and Sali, 2016), and the best model according to the PDF scoring function was selected for further simulations. The obtained model was subjected to a molecular minimization protocol using the CHARMM27 force-field using Discovery Studio v2.1 (Accerys Inc., San Diego, CA). The protocol consisted of 5,000 steps of steepest descent method followed by 10000 steps of conjugate gradient method to reach a final root-mean square (RMS) gradient of 0.001 kcal/mol Å. The overall quality of the final modeled structure was performed using the SAVES server (http://nihserver.mbi.ucla.edu/SAVES/).

Molecular docking was performed using OEDocking suite v4.3.4.1 (OpenEye, Cadence Molecular Sciences, Santa Fe, NM) (McGann, 2011).First, the receptor was prepared using the MakeReceptor GUI, and a box with dimensions 21x28x24 Å using the center of mass of the Lys05 ligand present in the template structure was defined. A site shape potential was set up, obtaining an outer contour of 3147 Å^3^; no constraints were defined. The ligands were constructed using MarvinSketch v23.3 (ChemAxon Ltd., www.chemaxon.com) and exported as df files. The stereocenters were enumerated using Flipper (part of OMEGA distribution) and multiconformer libraries of compounds were prepared using OMEGA v6.1.1.1 (OpenEye Scientific Software, Inc., Santa Fe, NM, USA) (Hawkins et al., 2010; OMEGA, 2025). QUACPAC v2.2.7.1 (OpenEye Scientific Software, Inc., Santa Fe, NM, USA) was used to assign AM1BCC charges to the library of conformers (QUACPAC, 2025). Finally, compounds were docked using FRED v4.3.4.1 (OpenEye Scientific Software, Inc., Santa Fe, NM, USA) and the best-scoring conformation according to the ChemGauss4 score of each compound was extracted for further analysis (McGann et al., 2003; FRED, 2025). The molecular interactions were calculated using the Protein-ligand interaction (PLIP) server (Schake et al., 2025). Predicted binding affinities were estimated using the PRODIGY-LIG web service, a variant specifically designed for protein–small-molecule complexes (Vangone et al., 2019). Graphical representations of docking poses were generated using PyMOL v3.1.6.1 (Schrödinger, LLC, Cambridge, MA, USA).

### 2.7 Data expression and statistical analysis

Data are expressed as mean ± SEM from at least 3–5 independent experiments. Concentration-response curves were analyzed by nonlinear regression to determine EC_50_ values and maximal responses (Emax). Statistical comparisons were performed using one-way ANOVA followed by Dunnett’s or Tukey’s *post hoc* test, or Student’s t-test where appropriate. For datasets that did not meet normality assumptions (Shapiro-Wilk test, p < 0.05), non-parametric analyses (Mann-Whitney U test) were applied. Correlation analysis used Pearson’s correlation coefficient (R^2^). p < 0.05 was considered statistically significant. Data analysis was performed using GraphPad Prism v9.0 (GraphPad Inc.).

## 3 Results

### 3.1 Aldosterone induces NO production in cultured endothelial cells

**FIGURE 2:**
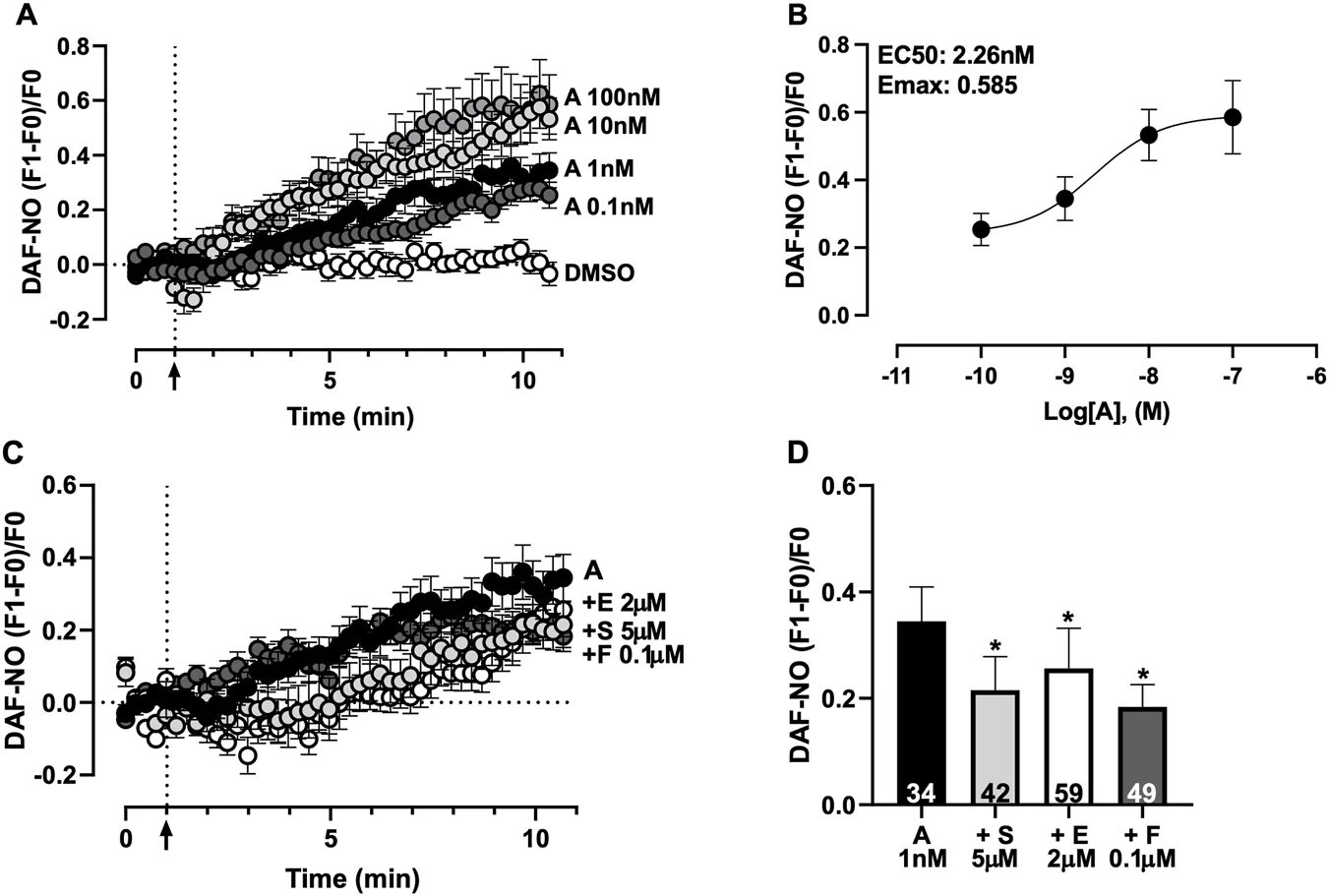
Aldosterone induces NO production as evidenced by increases in DAF-NO fluorescence; reduction in DAF-NO signaling by MR antagonists pretreatment. Panel **(A)** Time course rises of DAF-NO fluorescence signaling elicited by applications of 0.1, 1, 10 or 100 nM aldosterone **(A)** in representative separate single concentration protocols, as a negative control, 55 µM DMSO was used. Dashed lines represent the time of 1 nM aldosterone addition. Panel **(B)** Aldosterone NO production is concentration-dependent as evidenced by concentration-dependent rises in DAF-NO signaling; the EC_50_ derived from all protocols was 2.26 ± 0.3 nM, Emax = 0.585. Panel **(C)** Blockade of the aldosterone **(A)** induced DAF-NO signaling by hormone pretreatment with either 2 µM eplerenonev **(E)**, or 5 µM spironolactone (S) or 0.1 µM finerenone **(F)**. Panel **(D)** maximal inhibition of the aldosterone-induced DAF-NO signaling elicited in the presence of spironolactone (S, 5 μM, 42 cells examined), eplerenone (E, 2 μM, 59 cells examined) or pretreatment with 0.1 µM finerenone (F, 49 cells examined). In all graphs, symbols indicate mean values; error bars represent SEM. *p < 0.05, vs. vehicle control (one-way ANOVA with Tukey’s *post hoc* test).

In primary cultures of rat mesenteric endothelial cells, the hormone aldosterone consistently induced concentration-dependent increases in DAF-NO fluorescence signaling, verifying NO production (Figure 2A). The EC_50_ for NO production was 2.26 ± 0.3 nM (Figure 2B), with Emax of 245% ± 18% above baseline (n = 12).

**FIGURE 3:**
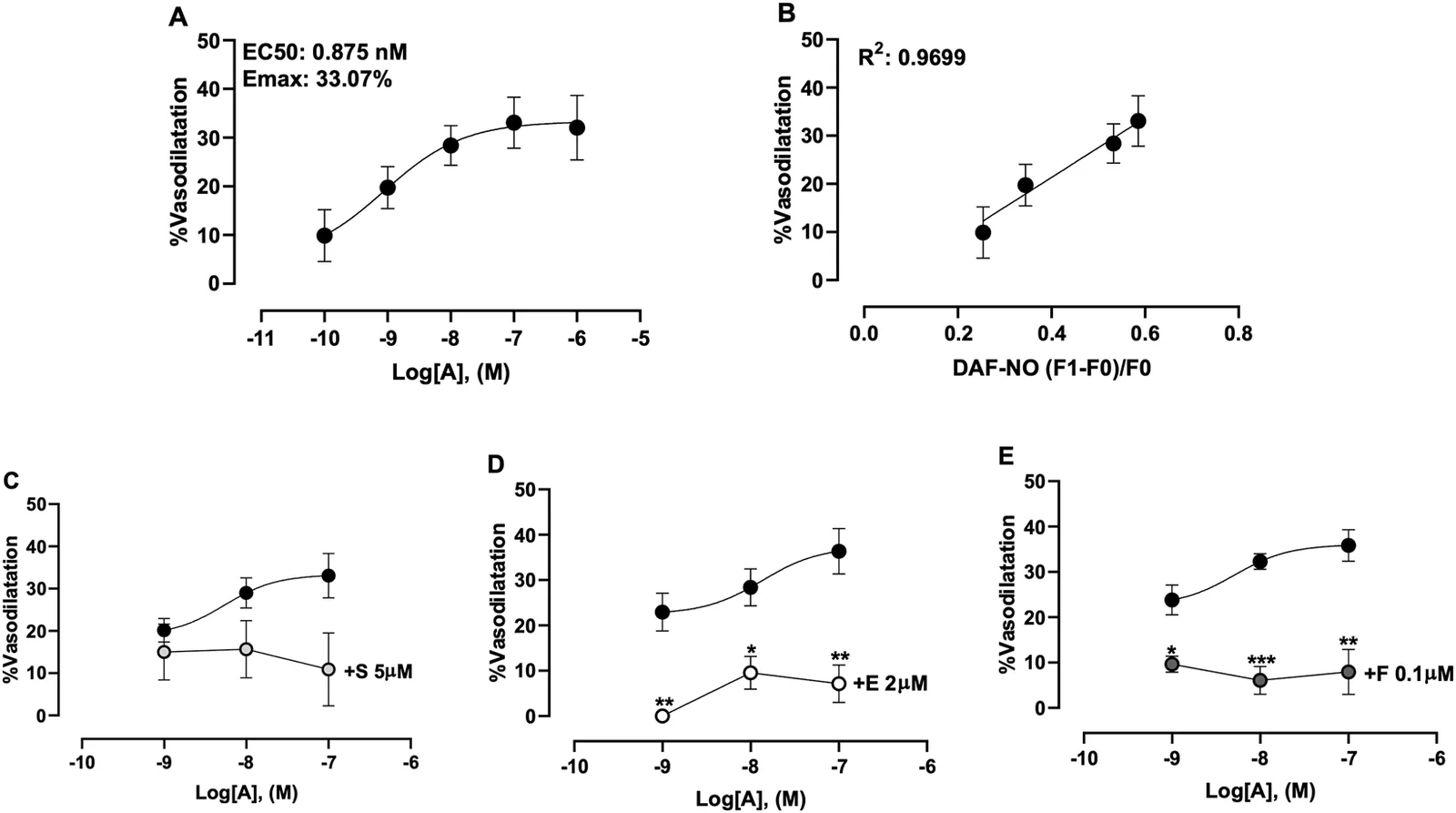
MR Antagonists reduce aldosterone-induced vasodilation. Panel **(A)** Concentration-response curve for aldosterone **(A)** induced vasodilation in NA-precontracted rat mesenteric arterial beds. EC_50_ = 0.9 ± 0.2 nM, Emax = 33% ± 5% of the maximal acetylcholine-induced response. Panel **(B)** Correlation between vasodilation and NO production elicited by varying aldosterone concentrations. Pearson correlation r^2^ = 0.969, p = 0.003. Panel **(C,D,E)** Aldosterone concentration-response curves in mesenteric beds precontracted with NA and pretreated with either spironolactone (5 μM, panel C), eplerenone (2 μM, panel D), or finerenone (100 nM, panel E). All 3 MR antagonists significantly reduced aldosterone-induced vasodilation and shifted aldosterone curves downwards. In all figures, symbols indicate mean values; error bars represent SEM, n = 4–5 independent preparations per MR antagonist examined. *,p < 0.05, **p < 0.01, ***p<0.001.

**FIGURE 4:**
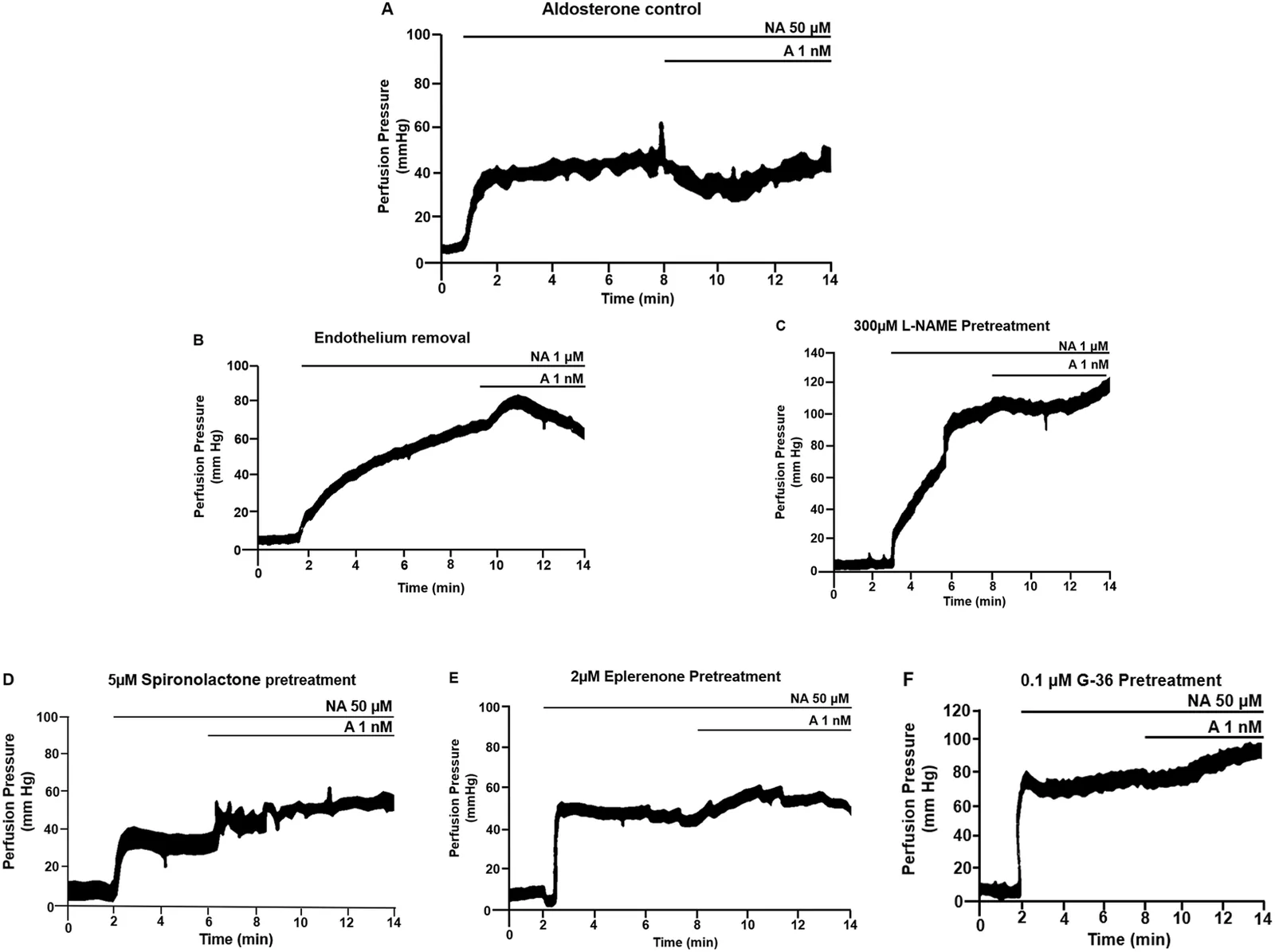
Representative aldosterone **(A)** induced vascular responses and its conversion to vasoconstriction by several mesentery pretreatments. Panel **(A)** Representative 1 nM aldosterone **(A)** perfusion to a rat mesentery preparation elicited transient vasodilatation of the rat mesenteric vascular bed. Panels **(B,C)** show that perfusion of the rat mesenteric bed with 1 nM aldosterone elicited transient vasoconstriction in representative mesentery preparations following either endothelium removal **(B)** or blockade of endothelial NO h activity by pretreatment with 300 μM L-NAME **(C)**, a recognized eNOS blocker. Panels **(D,E)** show that mesenteries pretreatment with either 5 uM spironolactone **(D)** or 2 uM eplerenone **(E)** elicited sustained aldosterone-induced vasoconstrictions. Furthermore, Panel **(F)** shows that following perfusion with 0.1 µM G-36, (a prototype GPER antagonist), aldosterone perfusion of the rat mesentery elicited a sustained vasocontractile response.

Aldosterone induced concentration-dependent vasodilation in noradrenaline-preconstricted rat mesenteric arterial beds (Figure 3A). The EC_50_ for aldosterone-induced vasodilation was 0.9 ± 0.2 nM, with a maximal response of 33% ± 5% (n = 8). The vasodilatation response was rapid, although transient reaching peak effect within 1–2 min; by 3–5 min base line values were recovered. A representative polygraphic tracing is shown in Figure 4A. Additional experiments showed that upon endothelium denudation (Figure 4B) or blockade of endothelial NO synthase following L-NAME pretreatment (Figure 4C), aldosterone did not cause vasodilation, but a short-lived vasocontractile response. These results suggest that the mesenteric vascular wall has both endothelium and smooth muscle aldosterone receptors, coupled to dilation and/or contractile vascular responses, respectively.

Correlation analysis revealed a strong positive relationship between aldosterone-induced NO production and its subsequent vasodilation along the concentration range tested (R^2^ = 0.969, p < 0.01, n = 8, Figure 3B), supporting NO as the primary mediator of aldosterone’s vasodilator effects in this vascular bed.

### 3.2 MR antagonists blocked the aldosterone responses but exhibit intrinsic activity

Endothelial cell preincubation with the MR antagonists 5 μM spironolactone, 2 μM eplerenone, or 0.1 μM finerenone significantly inhibited both the aldosterone-induced NO production (Figure 2C,D) as well as the subsequent mesentery-induced vasodilation (Figure 3C,D,E). Note that the finerenone concentration was 20 fold lower than eplerenone, indicating that finerenone is markedly more potent than the other MR antagonist to block the aldosterone vascular action. Interestingly and consistent with the endothelium denudation, or the occupation of endothelial MR receptors, by MR antagonists following perfusion of either 5 uM spironolactone (n = 4) or 2 uM eplerenone (n = 4), acute 1 nM aldosterone perfusion elicited vasocontractile responses, suggesting that the aldosterone endothelial MR receptor is sensitive to MR antagonists. In addition, upon occupation of the endothelial MR receptor by MR antagonists, aldosterone elicited short-lived vasomotor responses (Figure 4D,E) indicating that the presence of an aldosterone receptor in vascular smooth muscles. The latter aldosterone receptors are apparently not sensitive to MR receptor antagonists. Moreover, spironolactone reduced the 1 nM aldosterone-induced vasodilation from 62% ± 4% to 18% ± 3% (p < 0.001, n = 6), eplerenone reduced it to 24% ± 4% (p < 0.001, n = 6), and finerenone reduced it to 28% ± 5% (p < 0.001, n = 6), supporting the notion of endothelium MR receptor-mediated responses.

**FIGURE 5:**
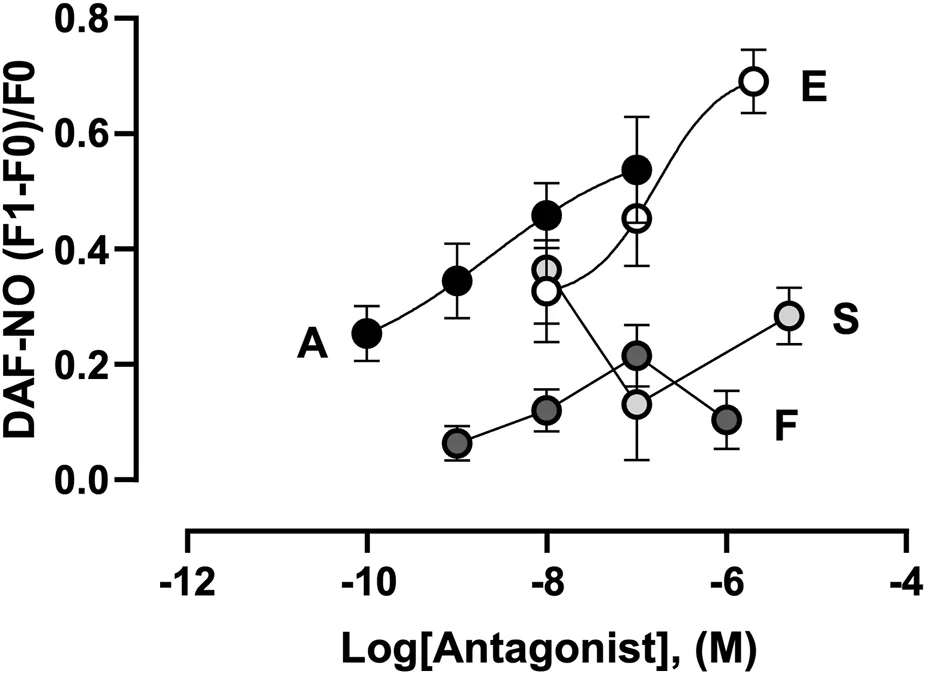
MR Antagonists produce *per se* NO, evidencing intrinsic GPER-linked agonist activity. DAF-FM fluorescence concentration-response curves of MR antagonists alone (without aldosterone) in cultured endothelial cells. Compared to 10 nM aldosterone the DAF-NO values for the MR antagonist at 10 nM were: spironolactone 79.5% ± 20.4%; eplerenone 82.9% ± 15.95%; and finerenone 25.9% ± 8.5%, respectively. All 3 MR antagonists produced concentration-dependent increases in DAF-NO signaling above baseline. When comparing the maximal intrinsic response of each antagonist against the aldosterone reference response by Mann-Whitney test, finerenone’s difference from baseline reached conventional significance (p < 0.0001); spironolactone and eplerenone responses, while numerically larger, showed greater inter-experiment variability. The concentration-dependent patterns of all three agents are nevertheless consistent with intrinsic agonist activity. Error bars represent SEM.

Unexpectedly, when tested in the absence of aldosterone, all 3 MR antagonists exhibited *per se* concentration-dependent NO production in endothelial cells (Figure 5), consistent with intrinsic agonist activity. The rank order of efficacy of the MR antagonists was eplerenone greater than spironolactone or finerenone. This intrinsic activity was concentration-dependent, consistent with receptor-mediated signaling rather than non-specific effects. The intrinsic NO-producing effect of MR antagonists was also observed in the isolated mesenteric bed preparation, where 10 μM spironolactone induced 35% ± 4% (n = 4) vasodilation relative to NA evoked vasoconstriction (n = 6), a value similar to that observed with aldosterone (Figure 3A). This result confirms that MR antagonist induced NO production and subsequent mesentery vasodilatation. A similar observation applies to eplerenone-induced mesentery vasodilatation (Supplementary Figure S1).

### 3.3 MR antagonist intrinsic activity is concentration-dependent and consistent with partial agonism

The intrinsic NO-producing activity of MR antagonists was concentration-dependent, occurring at concentrations between 10 and 1,000 nM (Figure 5). At clinically relevant concentrations (1–10 μM), all 3 MR antagonists exhibited measurable intrinsic activity, with eplerenone showing only 10-fold lower potency compared to aldosterone. Finerenone showed markedly less efficacy with blunted concentration-response curves (Figure 5). The coexistence of antagonist and intrinsic agonist activity—blockade of aldosterone responses at the same concentrations that produce *per se* NO signaling—is consistent with partial agonism at a common receptor site mediating NO production.

### 3.4 GPER mediates aldosterone-induced NO production and subsequent vasodilation

**FIGURE 6:**
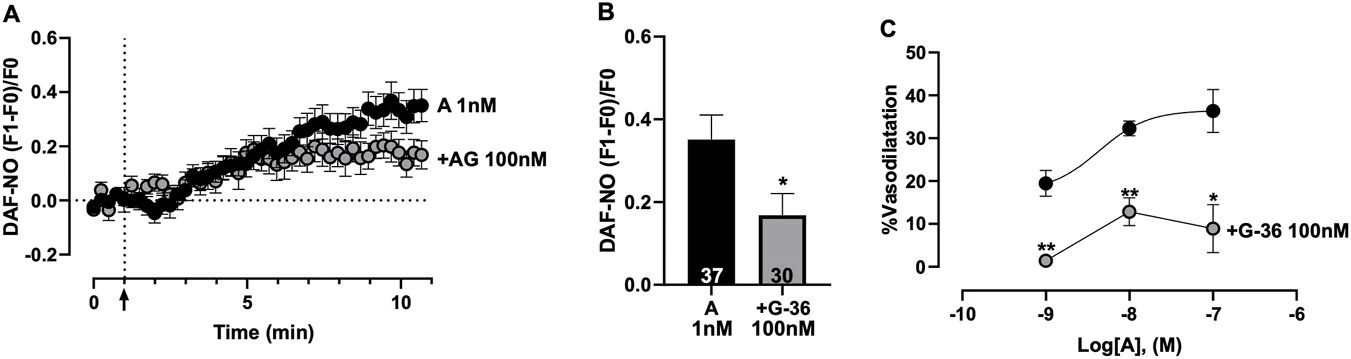
GPER participates in the aldosterone **(A)** induced NO production by endothelial cells and subsequent tissue vasodilation. Panel **(A)** The 1 nM aldosterone **(A)** induced DAF-NO signaling was reduced by endothelial cells pretreatment with 100 nM G-36 a recognized GPER antagonist. Dashed line and the arrow indicates the addition of 1 nM aldosterone to the cultured cells. Panel **(B)** Aldosterone induced NO production in endothelial cells pretreated with the hormone **(A)** or aldosterone plus G-36 (100 nM). The GPER antagonist significantly halved the aldosterone-induced DAF-NO fluorescence (64% inhibition). Panel **(C)** The aldosterone-induced concentration-dependent vasodilation was significantly reduced in mesenteries pretreated with 100 nM G-36, the GPER antagonist. Controls were treated/perfused with vehicle (55 µM DMSO). Symbols or columns denote mean values; error bars represent SEM of 5 averaged independent preparations. *, p <0.05, **, p<0.01.

To investigate the role of GPER in aldosterone’s rapid vascular effects, we used G-36 the selective GPER antagonist. In cultured endothelial cells, preincubation with 100 nM G-36, significantly inhibited aldosterone-induced NO production, reducing the response from 228% ± 15% to 98% ± 12% of baseline (p < 0.001, n = 10, Figure 6A,B). Importantly, G-36 also significantly reduced the intrinsic 5 μM spironolactone-induced DAF-NO signaling from 142% ± 11% to 88% ± 9% of baseline (p < 0.01, n = 8), suggesting that the partial agonist activity of MR antagonists is mediated, at least in part, through GPER. Consonant with the G-36 reduction of DAF-NO signaling elicited by 1 nM aldosterone, preincubation with 100 nM G-36 also significantly reduced the 1 nM aldosterone induced vasodilation from 64% ± 5% to 28% ± 4% (p < 0.001, n = 7, Figure 6C). G-36 alone had no effect on baseline vascular tone or the noradrenaline-induced constriction, confirming its selectivity as an antagonist without intrinsic vasodilator activity. Moreover, in a set of 4 additional mesentery preparations, perfusion with 100 nM G-36 not only obliterated the 1 nM aldosterone vasodilatation but rather we consistently observed in 4/5 experiments a vasomotor effect as illustrated in the representative tracing shown in Figure 4F.

### 3.5 GPER-EGFR transactivation contributes to aldosterone-induced NO production

**FIGURE 7:**
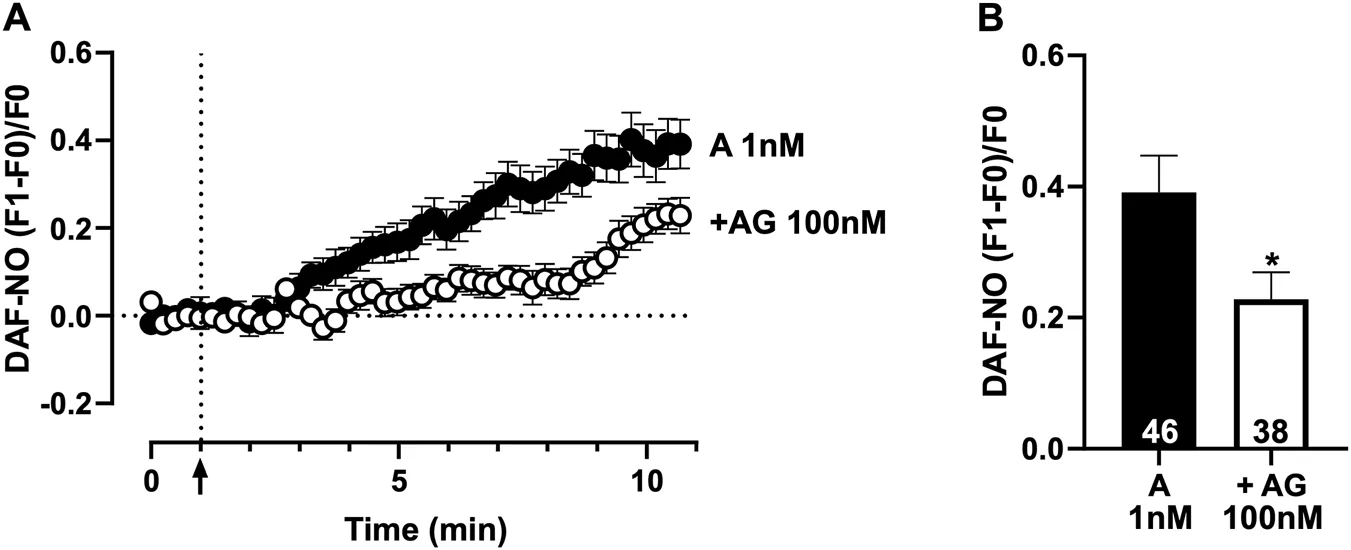
EGFR-GPER cross talk in aldosterone **(A)**-induced NO production Panel **(A)** Time-course of DAF fluorescence in endothelial cells treated with 1 nM aldosterone with or without pretreatment for 30 min of 100 nM AG-1478, an inhibitor of the EGFR phosphorylation. Dashed line and the arrow indicate the 1 nM aldosterone addition to cultured endothelial cells. Panel **(B)** Pretreatment with 100 nM AG-1478 significantly reduced peak DAF-NO fluorescence indicating reduced NO production (55% inhibition). Symbols indicate mean ± SEM, error bars represent SEM, n = 4 independent cell batches.

The EGFR inhibitor AG-1478 (100 nM) significantly reduced aldosterone (1 nM)-induced NO production in endothelial cells from 235% ± 18% to 132% ± 14% of baseline (p < 0.01, n = 9, Figure 7A,B), indicating GPER-EGFR transactivation contributes to aldosterone’s fast, non-genomic effects. Combined treatment with 100 nM G-36 plus 100 nM AG-1478 produced greater inhibition of aldosterone-induced NO production than either agent alone, reducing the response to 72% ± 8% of baseline (p < 0.001 vs. aldosterone alone, n = 8). This additive effect suggests that GPER and EGFR contribute through partially independent mechanisms to aldosterone’s rapid signaling, consonant with cross-talk mechanisms.

### 3.6 Intracellular signaling pathways implicated in aldosterone-induced NO production

**FIGURE 8:**
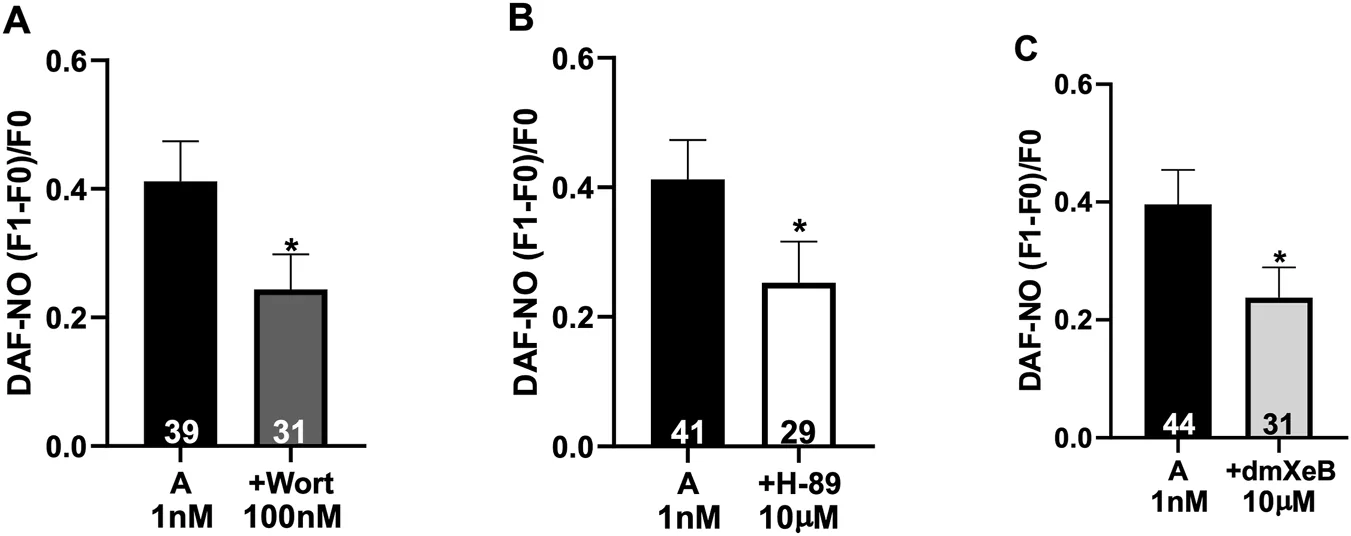
Intracellular signaling pathways required in aldosterone (A)-induced NO production. Panel **(A)** Effects of PI3K inhibitors on aldosterone-induced DAF fluorescence. Wortmannin (100 nM) significantly reduced by 45% the 1 nM aldosterone-induced DAF-NO signals. Panel **(B)** Pretreatment with 10 μM PKA inhibitor H89 reduced 40% the aldosterone-induced DAF fluorescence signal. Panel **(C)** Pretreatment with desmethyl xestospongin B (dmXeB, 10 μM) a recognized IP_3_ receptor antagonist significantly reduced the aldosterone-induced DAF fluorescence signal (37% inhibition). In all graphs, columns denote mean values; error bars represent SEM; n = 10 **(A)**, 9 **(B)** and 8 **(C)** independent cell preparations. *, *p < 0.05.*

To elucidate downstream signaling mechanisms, we examined the effects of selective pathway inhibitors on aldosterone-induced NO production, following a protocol similar to that previously reported by Catalán-Salas et al. (2024) to antagonize estrogen or phytoestrogens GPER signaling. 100 nM wortmannin, significantly reduced the 1 nM aldosterone-induced NO production from 242% ± 16% to 118% ± 11% of baseline (p < 0.001, n = 10, Figure 8A), indicating PI3K involvement. In a parallel set of experiments, LY294002 (a structurally distinct PI3K inhibitor) also significantly reduced aldosterone-induced NO production, further confirming the coupling of GPER to PI3K activity and ruling out off-target effects of wortmannin. Altogether, these results are consistent with PI3K participation in the rapid intracellular aldosterone-mediated responses. In a separate set of protocols, we examined whether cAMP is an intracellular messenger associated to GPER effects. 10 μM H-89, a well-known PKA inhibitor (Catalán-Salas et al., 2024) also significantly inhibited the response, reducing it to 135% ± 13% of baseline (p < 0.01, n = 9, Figure 8B). Finally, the IP_3_ receptor antagonist, desmethylxestospongin B (dmXeB, 10 μM) reduced the aldosterone-induced DAF-NO signaling to 128% ± 12% of baseline (p < 0.01, n = 8, Figure 8C), suggesting intracellular calcium mobilization in aldosterone in the activation of eNOS through NO production.

### 3.7 Computational modeling supports plausible GPER-engaging poses for aldosterone and MR antagonists at the rGPER

**FIGURE 9:**
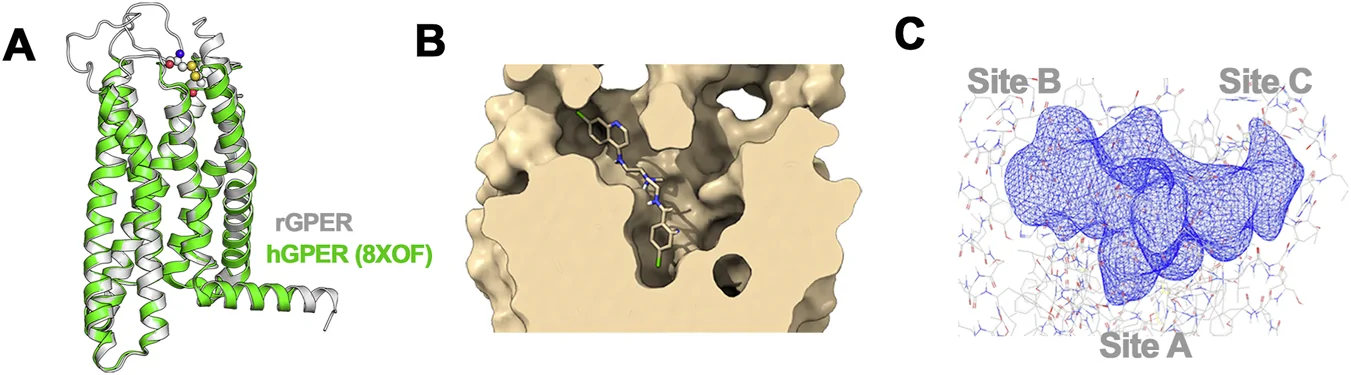
Comparative modeling of rat GPER, cavity detection, and binding-site definition based on the human GPER template. Panel **(A)** Structural alignment between the comparative model of rat GPER (rGPER, gray) and the human GPER cryo-EM template 8X0F (green), showing the overall conservation of the seven-transmembrane helical architecture used for model construction. The rGPER model was generated for residues 45–349 using Modeller, incorporating the Cys130–Cys207 disulfide bond, followed by energy minimization with the CHARMM27 force field (Supplementary Figure S2). Panel **(B)** Definition of the docking region and cavity organization. Surface representation of the receptor cavity surrounding the position of the co-crystallized template ligand, used to define the docking box in MakeReceptor. Panel **(C)** Mesh representation of the receptor inner volume highlighting the principal subcavities identified for docking and designated as sites A, B and C. The docking box was centered on the ligand present in the template structure and defined with dimensions of 21 × 28 × 24 Å, yielding a site-shape outer contour of 3147 Å^3^.Together, these analyses summarize the workflow used for model construction, cavity detection, and binding-site definition prior to docking calculations.

**FIGURE 10:**
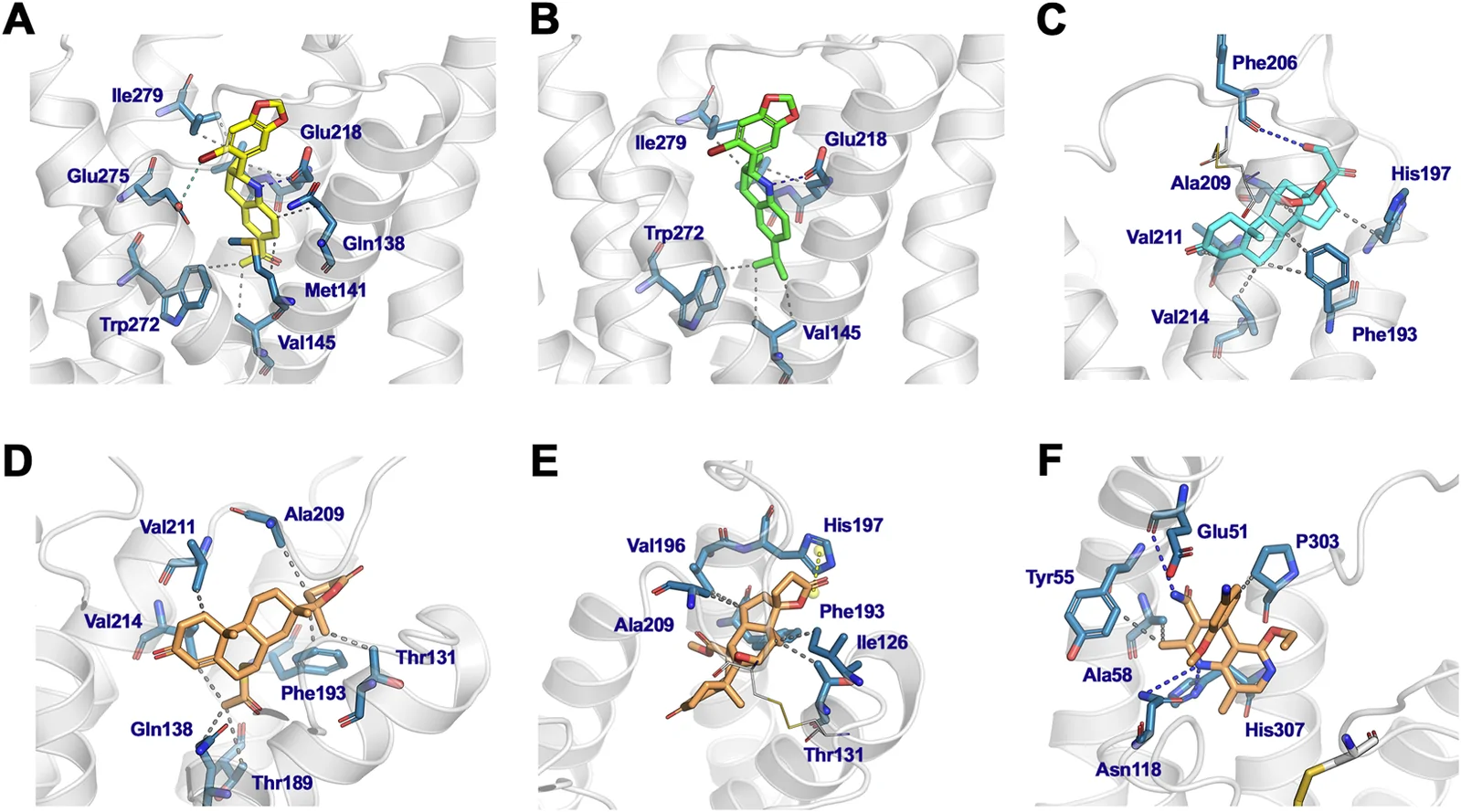
Docking analysis of aldosterone and mineralocorticoid receptor antagonists in the rGPER model. Representative docking poses and ligand–receptor interaction patterns obtained for reference GPER ligands and mineralocorticoid receptor ligands in the comparative model of rat GPER (rGPER). Panel **(A)** G-1 docked in site B, showing a binding mode stabilized predominantly by hydrophobic contacts with Gln138, Met141, Val145, Trp272, and Ile279, together with a hydrogen bond involving Glu218. Panel **(B)** G-36 docked in site B, occupying a closely related region and interacting with Val145, Glu218, Trp272, and Ile279, also through a hydrogen bond with Glu218. Panel **(C)** Aldosterone docked in site C, where the steroid nucleus is accommodated within a predominantly hydrophobic cavity lined by Phe193, His197, Ala209, Val211, and Val214, with an additional polar contact involving Phe206. Panel **(D)** Spironolactone docked in site C, adopting a pose partially overlapping that of aldosterone and interacting with Thr131, Gln138, Thr189, Phe193, Ala209, Val211, and Val214. Panel **(E)** Eplerenone docked in site C, sharing the same general pocket environment, with contacts involving Ile126, Thr131, Phe193, Val196, His197, and Ala209. Panel **(F)** Finerenone docked in site B, displaying a distinct orientation relative to the steroidal ligands and forming polar interactions with Glu51, Asn118, and His307, together with hydrophobic contacts with Tyr55, Ala58, and Pro303. Overall, the docking analysis consistently indicates that aldosterone, spironolactone, and eplerenone preferentially occupy a common cavity in site C, whereas finerenone favors an alternative binding region in site B, consistent with partially shared but non-identical modes of receptor recognition.

To explore whether the functional effects of aldosterone and the MR antagonists could be structurally compatible with GPER engagement, we performed docking analyses using a comparative rat GPER model and examined the resulting ligand poses and interaction patterns. Docking calculations performed on the comparative rGPER model showed that both reference GPER ligands and mineralocorticoid receptor ligands accommodate with favorable binding scores within recurrent cavities of the receptor (Figure 9). Among the compounds examined, 8XOF (Lys01) displayed the most favorable predicted binding energies, with ΔG values of −5.77 kcal/mol in site A/B and −5.68 kcal/mol in site A/C. The reference ligands G-1 and G-36 also showed stable docking solutions, with G-1 favoring site B (ΔG = −5.39 kcal/mol; Figure 10A) and G-36 adopting poses in sites A and B (ΔG = −5.09 and −5.31 kcal/mol, respectively; Figure 10B). Aldosterone yielded comparable binding energies across three cavities, with slightly more favorable values in sites A and B (ΔG = −5.42 and −5.43 kcal/mol) than in site C (ΔG = −5.39 kcal/mol), although the pose illustrated in the main figure highlights a site C orientation characterized by a hydrophobic environment formed by Phe193, His197, Ala209, Val211, and Val214 (Figure 10C). Spironolactone and eplerenone also docked favorably within this region, with spironolactone showing its best score in site C (ΔG = −5.74 kcal/mol; Figure 10D) and eplerenone with a ΔG = −5.38 kcal/mol in the same cavity (Figure 10E). In contrast, finerenone preferentially occupied site B (ΔG = −5.15 kcal/mol), where its predicted stabilization relied more strongly on polar contacts with Glu51, Asn118, and His307 (Figure 10F), while an alternative site C pose was also identified (ΔG = −5.18 kcal/mol). Additional calculations involving the several interactions with relevant GPER ligands are listed in Supplementary Table S1. Taken together, these data indicate that steroidal ligands tend to converge on a partially shared binding region, whereas the non-steroidal antagonist finerenone adopts a differentiated pose. However, the absolute ΔG differences among these ligands (∼0.6 kcal/mol) fall within the typical uncertainty of empirical docking scoring functions, and therefore the site preferences should be interpreted as plausible trends rather than definitive assignments of selectivity. These computational observations are consistent with the hypothesis that related pharmacological outcomes may arise from distinct receptor interaction patterns but require experimental validation through mutagenesis or direct binding studies.

## 4 Discussion

### 4.1 Principal findings

This study demonstrates using three complementary analytic techniques such as single-cell *in vitro* NO production, vascular reactivity bioassay and GPER bioinformatics that aldosterone induces rapid, NO-mediated vasodilation of the rat mesenteric bed through mechanisms involving GPER and EGFR signaling. We demonstrate for the first time that three clinically used MR antagonists (spironolactone, eplerenone, and finerenone) elicit endothelial NO production through a G-36-sensitive, GPER-linked pathway, consistent with intrinsic agonist activity at this signaling node. Both GPER and EGFR cross-talk through common signaling pathways that involve PI3K, PKA, and IP_3_ receptor pathways. These findings have significant implications for understanding the full pharmacological profiles of MR antagonists and their differential clinical effects, a finding not previously reported in the literature. Consonant with the proposal of aldosterone as a GPER ligand, bioinformatic calculations are consistent with docking sites with representative binding energies in several GPER cavities.

### 4.2 Aldosterone’s rapid vascular effects involve GPER activity

The significant inhibition of aldosterone responses by the GPER antagonist G-36 provides direct evidence for GPER involvement in aldosterone’s rapid vascular signaling. This finding aligns with previous reports implicating GPER in rapid steroid effects (Revankar et al., 2005; Prossnitz and Barton, 2011), and extends the range of steroid hormones known to signal through GPER beyond estrogens to include aldosterone and MR antagonists as well.

**FIGURE 11:**
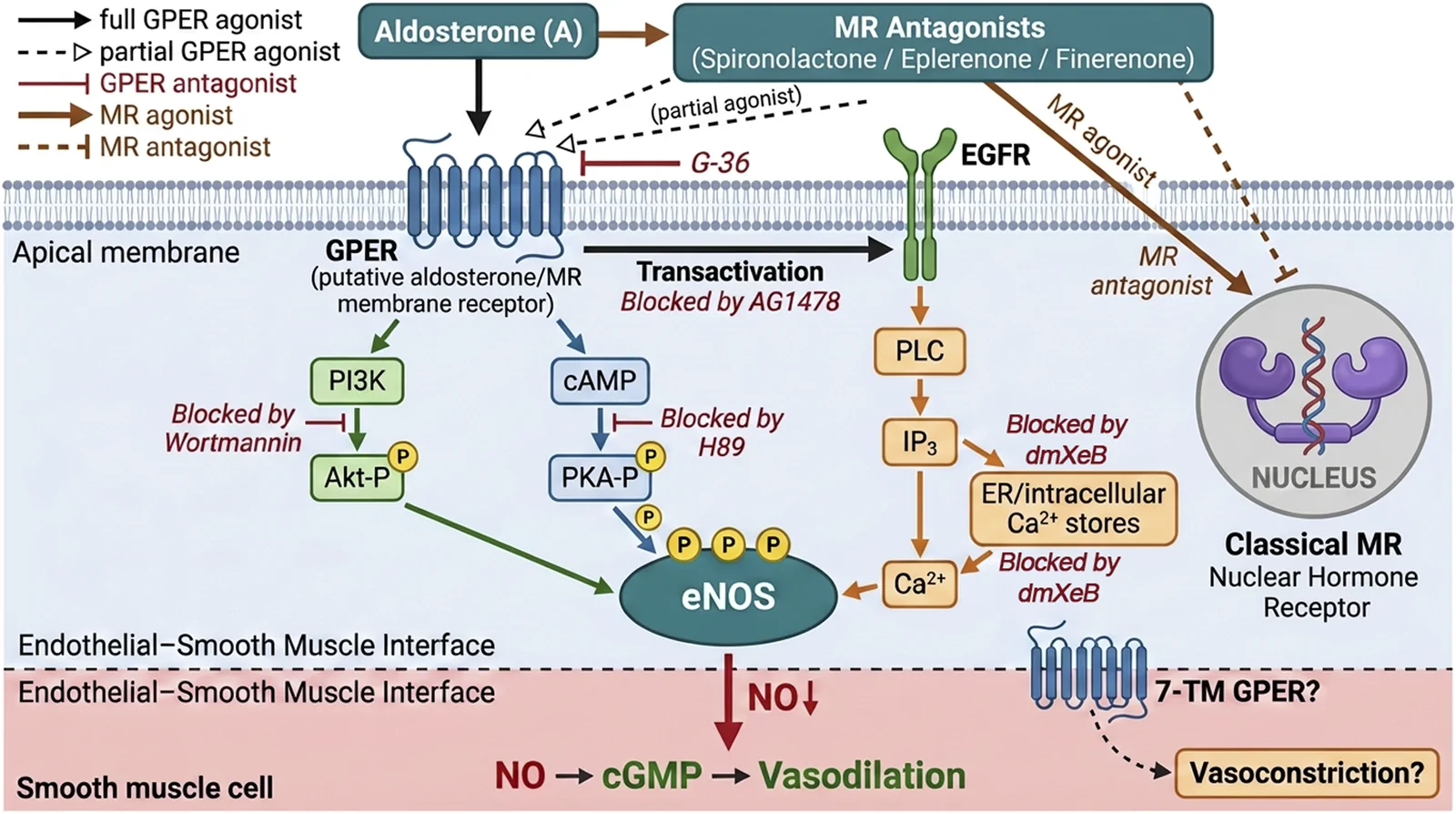
Proposed heuristic model of aldosterone (A) fast, non-genomic response leading to NO production through GPER signaling in endothelial cells and sites for MR antagonists as GPER-linked agonists. The apical membrane of the endothelial cell depicts GPER as the putative aldosterone membrane receptor mediating non-genomic vascular responses, and its transactivation of EGFR (blocked by AG1478). Aldosterone (A; full GPER agonist, solid arrow) and MR antagonists—spironolactone, eplerenone, and finerenone (partial GPER agonists, dashed arrows) — act on GPER from the extracellular space. G-36, a selective GPER antagonist, blocks GPER activation (red flat-headed arrow) without interacting with the classical MR. Aldosterone additionally acts as an MR agonist (solid brown arrow) at the classical nuclear mineralocorticoid receptor (MR), a nuclear hormone receptor dimer that regulates gene transcription; MR antagonists competitively inhibit this genomic pathway (dashed brown flat-headed arrows). GPER activation leads to three parallel intracellular signaling branches converging on eNOS phosphorylation: (i) EGFR transactivation → PI3K → Akt (blocked by wortmannin); (ii) cAMP → PKA (blocked by H89); and (iii) PLC → IP_3_ → ER Ca^2+^ release → Ca^2+^ (blocked by dmXeB). Phosphorylation of eNOS at multiple sites drives NO production, which diffuses across the endothelial–smooth muscle interface to activate guanylyl cyclase, elevate cGMP, and elicit vasodilation. A putative GPER variant in the smooth muscle cell membrane may additionally mediate vasoconstriction. The temporal sequence of events and full pathway architecture require further experimental validation.

Current findings confirm and extend previous reports that aldosterone induces rapid, NO-dependent vasodilation (Liu et al., 2003; Mutoh et al., 2008). The strong correlation between vasodilation and endothelial NO production (R^2^ = 0.969) established NO as the primary mediator of aldosterone’s acute vascular effects in the mesenteric circulation. The rapid time course (2–3 min) and insensitivity to transcriptional inhibitors, consistent with previous reports of aldosterone rapid vascular effects being refractory to actinomycin D and cycloheximide (Liu et al., 2003; Heylen et al., 2009), confirm the non-genomic nature of the vascular response. Moreover, results also reveal a vasomotor effect of aldosterone, observed upon obliteration of the endothelial vasodilator response, likely due to an aldosterone receptor tentatively localized in smooth muscle. This dual vascular activity is fully compatible with the short-lived, aldosterone-induced vasodilator response. This hypothesis is schematized in Figure 11, showing GPER both in the endothelial cell as well as the vascular smooth muscle. As to whether the aldosterone receptor in the endothelium and in the smooth muscle is the same remains to be determined.

#### 4.2.1 Structural context of GPER-aldosterone interactions

Recent structural and pharmacological studies provide a novel and compelling framework to interpret the role of GPER in aldosterone signaling. The cryo-EM structure of GPER reported by Kaneda et al. (2024) revealed unique extracellular pockets that accommodate bicarbonate ions rather than classical deep steroid-binding cavities. Complementary functional studies demonstrated that bicarbonate signaling through GPR30 regulates vascular ischaemia-reperfusion injury *in vivo* (Jo-Watanabe et al., 2024), establishing the physiological significance of this ion-sensing mechanism. This structural architecture, combined with evidence from Liu et al. (2024) that estrogen is unlikely to be GPER\'s direct cognate ligand, initially suggested that GPER may not bind aldosterone directly in a classical steroid-receptor mode.

However, definitive radioligand binding evidence has now established aldosterone as a direct GPER agonist, with a Ki of 22 nM (95% CI: 4.1–100 nM) and an IC_50_ of 0.09 nM in competition binding (Ding et al., 2022). Critically, the use of insect cells lacking endogenous mineralocorticoid receptors eliminated potential confounding MR-mediated transactivation, providing unequivocal evidence for direct aldosterone-GPER interaction. The correlation between functional potency and binding affinity, together with GppNHp-induced reduction of radioligand binding (46%), confirmed a G-protein-coupled mechanism. These findings reconcile the apparent structural paradox: although GPER’s extracellular architecture differs from classical steroid receptors, the receptor nevertheless accommodates aldosterone with high affinity, possibly through binding sites that do not overlap with the bicarbonate-responsive pocket A.

Given both the direct binding evidence and the non-canonical receptor architecture, several complementary mechanisms may operate in aldosterone-GPER signaling: (1) aldosterone may bind to alternative sites on GPER (e.g., pockets B or C) distinct from the principal bicarbonate-responsive region; (2) membrane-proximal effects of aldosterone on lipid microdomains may modulate receptor conformation and facilitate ligand access; (3) GPER may function as part of a multi-component signaling complex in which direct binding and protein-protein interactions synergize; or (4) the promiscuous ligand recognition of GPER, which also accommodates natural products such as oleuropein and hydroxytyrosol (Chimento et al., 2014), may reflect structural flexibility enabling diverse ligand accommodation.

The identification of specific residues critical for bicarbonate binding and GPER activation (Kaneda et al., 2024), together with the divergent G-protein coupling geometry compared to other class A GPCRs, indicates that GPER functions as a receptor with unique activation mechanisms. This structural and pharmacological framework is fully consistent with our functional data showing GPER antagonism sensitivity and supports a model in which GPER mediates aldosterone\'s rapid vascular effects through direct receptor engagement. GPER has accordingly been proposed as a promising therapeutic target for aldosterone-induced hypertension (Li et al., 2023).

### 4.3 Aldosterone signaling involves GPER-EGFR cross-talk

The partial inhibition of aldosterone responses by the EGFR phosphorylation inhibitor, AG-1478, and the additive effects of combined GPER plus EGFR blockade, demonstrate that aldosterone activates both GPER and EGFR in endothelial cells, highlighting active cross-talk mechanisms. This is consistent with the well-established GPER-EGFR transactivation mechanism involving metalloproteinase-mediated heparan-bound EGF (HB-EGF) release (Filardo et al., 2000; Prossnitz et al., 2008). Our data consistently shows that aldosterone, acting through GPER, triggers EGFR transactivation, which contributes to downstream PI3K/Akt activation and eNOS phosphorylation and enzyme activation causing subsequent NO production.

The involvement of both GPER and EGFR provides a mechanistic explanation for the complexity of aldosterone’s rapid signaling and may account for tissue-specific differences in aldosterone responses. The relative contributions of GPER-direct and GPER-EGFR-mediated signaling likely depend on receptor expression levels, availability of metalloproteinases and HB-EGF, and the specific complement of downstream signaling molecules in different vascular beds.

### 4.4 Intrinsic GPER-linked agonist activity of MR antagonists

The most novel finding of this study is that spironolactone, eplerenone, and finerenone show intrinsic activity in endothelial NO production in the absence of aldosterone. This intrinsic activity is concentration-dependent and sensitive to GPER antagonism, indicating that MR antagonists can activate GPER-EGFR signaling pathways without aldosterone.

Partial agonism is a well-recognized pharmacological mode of drug action in which a ligand classified as an antagonist at one receptor exhibits agonist activity at another receptor or in a different signaling context (Mailman, 2007; Kenakin, 2014; Donoso et al., 2025). In the case of MR antagonists, their primary therapeutic action is competitive antagonism at the classical MR, blocking aldosterone\'s genomic effects on sodium retention. However, our data reveal that these compounds also activate a GPER-linked signaling pathway, producing submaximal activation of rapid, non-genomic signaling. Recent radioligand binding studies have demonstrated that aldosterone binds directly to GPER (Ki = 22 nM), supporting the interpretation that MR antagonists may similarly engage GPER directly (Ding et al., 2022). Whether the partial agonist activity of MR antagonists at GPER involves the same binding site as aldosterone or an allosteric mechanism remains to be fully elucidated.

#### 4.4.1 Molecular basis of MR antagonist-GPER interactions

The structural basis for MR antagonist interactions with GPER can be informed by recent advances in GPER structural biology and computational modeling. Pioneering homology modeling and molecular dynamics simulations established the three-dimensional framework for understanding GPER-ligand interactions, identifying key binding residues and characterizing agonist versus antagonist binding modes through 120-ns molecular dynamics trajectories (Méndez-Luna et al., 2015). It was further demonstrated dual binding modes for GPER agonists using comparative homology models of inactive and active receptor states, identifying critical helix movements and toggle-switch mechanisms underlying receptor activation (Arnatt and Zhang, 2013). Although direct structural data for MR antagonist-GPER complexes are not yet available, these computational studies, together with more recent docking analyses, have provided insights into ligand-GPER interactions. Lu et al. (2023) demonstrated that GPER residues Arg, Glu, His, and Asn form hydrogen bonds with hydroxyl, ether, or amine groups of ligands. The synthetic GPER agonist G-1 forms hydrogen bonds with Glu121, while computational studies by Elamin et al. (2024) identified Arg253 and Arg254 as key residues for G-1 and LNS8801 binding (Lu et al., 2023; Elamin et al., 2024).

The steroidal structures of spironolactone and eplerenone, and the non-steroidal structure of finerenone, all contain polar functional groups (lactone rings, ketones, hydroxyl groups) capable of forming hydrogen bonds with GPER residues. The rank order of MR antagonist’s intrinsic efficacy may reflect differences in binding affinity, binding mode, or efficacy of receptor activation related to these structural features. Spironolactone’s 17α-thioacetyl group and eplerenone’s 9α,11α-epoxy bridge may confer distinct interactions with the GPER binding pocket compared to finerenone non-steroidal scaffold.

Given the recent structural evidence that GPER has bicarbonate-binding pockets rather than classical steroid-binding sites (Liu et al., 2024; Kaneda et al., 2024), the mechanism by which steroidal MR antagonists activate GPER likely differs from classical steroid-receptor interactions. Possible mechanisms can include: (1) binding to alternative sites on GPER distinct from the bicarbonate pocket; (2) allosteric modulation of GPER through interactions with extracellular loops or transmembrane domains; (3) indirect effects on GPER through modulation of membrane properties or associated proteins; or (4) promotion of conformational changes that favor G-protein coupling independent of orthosteric ligand binding. The elucidation of these mechanisms awaits further bioinformatic advances.

Future structural studies, including cryo-EM structures of GPER in complex with MR antagonists or structure-guided mutagenesis experiments, will be necessary to definitively establish the molecular basis of MR antagonist-GPER interactions and to determine whether these compounds bind to the same sites as synthetic GPER agonists like G-1 and LNS8801.

### 4.5 Clinical implications of GPER-linked intrinsic activity of MR antagonists

#### 4.5.1 Differential clinical efficacy

The rank order of intrinsic GPER-linked activity of MR antagonists (eplerenone ≥ spironolactone > finerenone) may contribute to differences in clinical efficacy and side effect profiles among these agents. The greater GPER-linked intrinsic activity of eplerenone and spironolactone could contribute to cardiovascular benefits beyond MR blockade, potentially explaining why these steroidal MR antagonists have shown robust mortality benefits in heart failure trials (Pitt et al., 1999; Pitt et al., 2003). The additional GPER-linked activity might also contribute to spironolactone’s higher incidence of gynecomastia and other endocrine side effects, given the known role of GPER in estrogen-responsive tissues.

Finerenone, with the lowest intrinsic GPER-linked activity in our study, has demonstrated distinct clinical effects compared to steroidal MR antagonists, including greater efficacy in reducing albuminuria and cardiovascular events in diabetic kidney disease (Bakris George et al., 2020). Whether finerenone’s reduced GPER-linked agonism contributes to its unique clinical profile warrants investigation.

#### 4.5.2 Vascular protection

The ability of MR antagonists to stimulate endothelial NO production through GPER may contribute to vascular protective effects. NO is a critical regulator of vascular homeostasis, promoting vasodilation, inhibiting platelet aggregation, preventing leukocyte adhesion, and suppressing smooth muscle proliferation (Förstermann and Sessa, 2012). The GPER-mediated NO production by MR antagonists may provide additional cardiovascular benefits beyond blocking aldosterone’s deleterious genomic effects, including improved endothelial function, reduced vascular inflammation, and enhanced vascular remodeling.

#### 4.5.3 Sex differences in drug response

GPER expression and signaling exhibit sex differences in various tissues (Xu et al., 2023). The partial agonist activity of MR antagonists at GPER could contribute to sex-specific differences in drug efficacy and tolerability. Clinical trials have reported sex differences in response to MR antagonists (Merrill et al., 2019), and GPER-mediated effects may partially explain these observations. Future studies should investigate whether GPER genotype or expression levels predict individual responses to MR antagonist therapy.

#### 4.5.4 Drug development

Recognition of MR antagonist partial agonism at GPER should inform development of next-generation MR antagonists. Depending on therapeutic goals, GPER activity could be either enhanced (to maximize vascular protection) or minimized (to reduce off-target effects). Structure-activity relationship studies guided by GPER structural data and computational modeling could enable rational design of MR antagonists with tailored GPER activity profiles (Lu et al., 2023; Elamin et al., 2024; Liu et al., 2024; Kaneda et al., 2024).

The clinical development of selective GPER agonists, exemplified by LNS8801, provides a complementary approach to exploiting GPER’s therapeutic potential. LNS8801’s enantiomeric purity, established by single-crystal X-ray analysis, ensures selective GPER activation without MR antagonism (Elamin et al., 2024; Natale et al., 2025). The demonstration that LNS8801 induces mitotic arrest, upregulates p53/p21, promotes differentiation, and suppresses tumor growth in preclinical models highlights GPER’s potential as a therapeutic target beyond cardiovascular disease. The advancement of LNS8801 into clinical trial represents an important milestone in translating GPER structural and pharmacological insights into therapeutic applications.

### 4.6 Signaling mechanisms

Our pathway analysis revealed that aldosterone-induced NO production directly involves PI3K, PKA, and IP_3_ receptor signaling. These pathways converge on eNOS activation through multiple mechanisms: PI3K/Akt-mediated phosphorylation of eNOS at Ser1177 (Lu et al., 2023), PKA-mediated phosphorylation at Ser1177 and Ser633 (Filardo et al., 2000), and calcium-calmodulin binding following IP_3_-mediated calcium release (Förstermann and Sessa, 2012). The involvement of multiple parallel pathways provides redundancy and signal amplification, ensuring robust NO production in response to aldosterone.

The similar sensitivity of MR antagonist-induced NO production to these pathway inhibitors supports the conclusion that MR antagonists activate the same signaling networks as aldosterone, consistent with their partial agonist activity at GPER. The GPER-EGFR-PI3K/PKA-eNOS signaling axis represents a rapid, non-genomic pathway that complements aldosterone’s classical genomic effects on gene transcription.

### 4.7 Receptor reserve and tissue context

The partial agonist activity of MR antagonists was observed at concentrations (1–10 μM) that are clinically relevant. Plasma concentrations of spironolactone and its active metabolites reach 1–3 μM with standard dosing (Gardiner et al., 1989), while eplerenone plasma concentrations reach 2–5 μM (Cook et al., 2003). Finerenone plasma concentrations are lower (0.1–0.5 μM) but tissue concentrations may be higher due to lipophilicity and tissue accumulation (Heinig et al., 2016).

The magnitude of intrinsic agonist effects depend on receptor reserve, the fraction of receptors that must be occupied to produce a maximal response (Kenakin, 2014). In tissues with high GPER expression and large receptor reserve, agents with intrinsic activity may produce near-maximal responses. Conversely, in tissues with low receptor reserve, they produce submaximal effects. This context dependence may explain tissue-specific differences in MR antagonist effects and highlights the relevance of considering receptor expression levels when predicting drug responses.

### 4.8 Limitations and future directions

Several limitations of this study should be acknowledged. First, experiments were conducted in rat tissues and cells; species differences in GPER expression, signaling, and pharmacology may limit direct extrapolation to human cardiovascular outcomes. Although GPER is highly conserved across mammals, receptor density, coupling efficiency, and downstream signaling fidelity may differ between rat mesenteric endothelium and human coronary or systemic vascular beds. Second, we used pharmacological antagonists to implicate GPER and EGFR; genetic approaches (siRNA, CRISPR/Cas9) would provide more definitive evidence of receptor identity. Third, we focused on NO production as the primary endpoint; MR antagonists may exhibit intrinsic activity for other GPER-mediated responses (calcium mobilization, MAPK activation, gene transcription) that warrant separate investigation. Fourth, the GPER-dependence of intrinsic activity was directly tested only for spironolactone (G-36 sensitivity); extending this test to eplerenone and finerenone is required before the GPER-linked mechanism can be generalized with confidence to all three agents. Fifth, the translation of these *in vitro* endothelial findings to systemic human hemodynamics requires validation in human endothelial cell lines (e.g., HUVECs, human coronary artery endothelial cells) and ultimately in clinical cohorts where GPER expression and MR antagonist tissue concentrations can be measured concurrently.

Future studies should: (1) determine whether MR antagonist partial agonism at GPER occurs in human endothelial cells and other cell types; (2) investigate the structural basis of MR antagonist-GPER interactions using computational modeling, site-directed mutagenesis, and, ideally, cryo-EM structural determination of GPER-MR antagonist complexes; (3) assess whether GPER genotype or expression levels predict individual responses to MR antagonist therapy in clinical populations (4) evaluate whether selective GPER agonists (e.g., G-1, LNS8801); or antagonists (e.g., G-36) modulate the cardiovascular effects of MR antagonists *in vivo*; and (5) determine whether next-generation MR antagonists can be designed with optimized GPER activity profiles for specific therapeutic applications.

The recent structural breakthroughs in GPER biology provide a foundation for structure-guided drug design. Integration of cryo-EM structural data, computational modeling, and functional pharmacology will enable rational optimization of GPER modulators with improved selectivity, potency, and pharmacokinetic properties. The successful clinical development of LNS8801 demonstrates the feasibility of this approach and provides a roadmap for translating GPER structural insights into therapeutic applications.

### 4.9 Final conclusions

This study demonstrates that MR antagonists elicit endothelial NO production through a G-36-sensitive, GPER-linked pathway functionally coupled to EGFR signaling. This finding expands our understanding of MR antagonist pharmacology beyond classical MR blockade and may contribute to explaining differential clinical efficacy and side effect profiles among these agents. Recognition of MR antagonist-GPER interactions, informed by recent structural advances in GPER bioinformatics, should guide development of next-generation MR antagonists and selective GPER modulators with optimized therapeutic profiles. The convergence of structural biology, computational modeling, and functional pharmacology promises to accelerate rational design of cardiovascular therapeutics targeting GPER and related signaling pathways.

## Data Availability

The raw data supporting the conclusions of this article will be made available by the authors, without undue reservation, on request to the corresponding author.
