# Supplementary material for: GPER and EGFR cross-talk highlight aldosterone- and MR antagonist-induced NO production in cultured endothelial cells
Source: Front Pharmacol. 2026 Sep 1;17:1891002. doi: 10.3389/fphar.2026.1891002 (PMC13575614; doi:10.3389/fphar.2026.1891002)
Supplement: Supplementary file 1 [file Supplementaryfile1.docx]

**Supplementary Figure S1.** Eplerenone-induced vasodilatation in isolated rat mesenteric arteries. Vascular tension recordings from endothelium-intact third-order mesenteric arteries pre-contracted with phenylephrine (1 µM). Eplerenone (2 µM, red solid line) produced a sustained vasodilatation compared with the reference response to aldosterone (1 nM, blue dashed line). Data are expressed as percentage change in vascular tone from baseline (drug added at time 0) and presented as mean ± SEM (shaded bands).

**Supplementary Figure S2.** Sequence alignment of the template structure (8XOF), human GPER, and the modeled rGPER construct, illustrating the overall conservation of transmembrane segments and the structural basis for comparative model generation.

**Supplementary Table S1. Docking scores and estimated binding free energies for reference GPER ligands, aldosterone, and mineralocorticoid receptor antagonists in the rGPER model.**

| **Ligand** | **FRED Chemgauss4 score** | **Pose Index** | **CG3:Steric** | **CG3:Clash** | **CG3:ProDesolv** | **CG3:LigDesolv** | **CG3:LigDesolvHB** | **CG4:HB** | **Site** | **ΔGbind (kcal/mol)** |
| --- | --- | --- | --- | --- | --- | --- | --- | --- | --- | --- |
| Lys01 | -13.128 | 1 | -18.956 | 0.445 | 2.065 | 4.410 | -1.029 | -0.062 | A/B | -5.77 |
| Lys01 | -12.161 | 20 | -13.192 | 0.213 | 2.587 | 3.858 | -3.596 | -2.031 | A/C | -5.68 |
| G36 | -11.810 | 1 | -15.243 | 0.443 | 2.258 | 1.314 | -0.395 | -0.185 | A | -5.41 |
| G1 | -10.637 | 1 | -14.569 | 0.493 | 2.177 | 1.845 | -0.395 | -0.188 | B | -5.39 |
| Spiro | -9.358 | 1 | -13.384 | 0.599 | 2.396 | 1.304 | -0.273 | 0.000 | A | -5.44 |
| Spiro | -8.694 | 5 | -12.977 | 0.668 | 2.825 | 1.303 | -0.205 | -0.306 | C | -5.74 |
| Fine | -8.665 | 1 | -13.078 | 0.442 | 3.023 | 2.716 | -0.811 | -0.958 | B | -5.15 |
| Fine | -8.531 | 4 | -12.020 | 0.554 | 1.924 | 3.265 | -1.049 | -1.205 | C | -5.18 |
| Eple | -8.779 | 1 | -12.764 | 0.656 | 1.881 | 1.359 | -0.053 | 0.000 | B | -5.38 |
| Eple | -6.792 | 98 | -10.329 | 0.396 | 1.417 | 1.938 | -0.215 | 0.000 | C | -5.74 |
| Aldo | -8.110 | 1 | -12.559 | 0.456 | 1.922 | 2.644 | -0.218 | -0.609 | C | -5.39 |
| Aldo | -7.887 | 24 | -12.585 | 0.556 | 3.048 | 2.232 | -0.385 | -0.779 | B | -5.43 |

The table summarizes the top-ranked docking poses obtained for Lys01, G-36, G-1, aldosterone, spironolactone (Spiro), eplerenone (Eple), and finerenone (Fine) using the FRED docking protocol. For each selected pose, the Chemgauss4 score and its individual energetic terms are shown, including steric complementarity (CG3:Steric), steric clashes (CG3:Clash), protein desolvation (CG3:ProDesolv), ligand desolvation (CG3:LigDesolv), ligand desolvation hydrogen-bond contribution (CG3:LigDesolvHB), and hydrogen-bonding contribution (CG4:HB). The pose index identifies the ranked docking solution selected for analysis, and the assigned site indicates the receptor cavity in which the ligand was located (A, B, C, or shared cavity regions such as A/B and A/C). The estimated binding free energy (ΔGbind, kcal/mol) is included as a complementary metric of predicted ligand stabilization. More negative Chemgauss4 and ΔGbind values indicate more favorable predicted receptor engagement. Overall, the results show that multiple ligands can be accommodated in distinct extracellular cavities of the rGPER model, with steroidal ligands tending to occupy partially overlapping regions and finerenone exhibiting a comparatively differentiated docking pattern.

**Supplementary Table S2.** Alignment of signaling-pathway targets, selective inhibitors, and measured baseline/residual NO metrics. NO signals are expressed as % of baseline DAF-NO fluorescence; residual = signal remaining after inhibitor pre-incubation in the presence of 1 nM aldosterone (mean ± SEM).

| **Pathway target** | **Selective inhibitor** | **Concentration** | **Mechanism/Selectivity** | **Residual NO (% Baseline)** |
| --- | --- | --- | --- | --- |
| PI3K / Akt | Wortmannin | 100nM | Covalent p110 (PI3K) inhibitor | 118 ± 11 (p < 0.001, n = 10) |
| PI3K / Akt | LY294002 | 10 µM | Reversible ATP-site PI3K inhibitor (confirmatory) | Reduced (data not shown) |
| PKA (cAMP) | H-89 | 10 µM | ATP-competitive PKA inhibitor | 135 ± 13 (p < 0.01, n = 9) |
| IP3R / Ca2+ | desmethylxestospongin B (dmXeB) | 10 µM | Selective IP3-receptor blocker | 128 ± 12 (p < 0.01, n = 8) |
| GPER | G-36 | 100 nM | Selective GPER antagonist | 98 ± 12 (p < 0.001, n = 10) |
| EGFR | AG-1478 | 100 nM | EGFR tyrosine-kinase inhibitor | 132 ± 14 (p < 0.01, n = 9) |
